# Study of Cardiovascular Risk Factors in Adolescents (ERICA): results and potentiality

**DOI:** 10.1590/S01518-8787.201605000SUPL1AP

**Published:** 2016-02-02

**Authors:** Katia Vergetti Bloch, Marly Augusto Cardoso, Rosely Sichieri

**Affiliations:** I Instituto de Estudos em Saúde Coletiva. Universidade Federal do Rio de Janeiro. Rio de Janeiro, RJ, Brasil; II Departamento de Nutrição. Faculdade de Saúde Pública. Universidade de São Paulo. São Paulo, SP, Brasil; III Departamento de Epidemiologia. Instituto de Medicina Social. Universidade do Estado do Rio de Janeiro. Rio de Janeiro, RJ, Brasil

This supplement presents 13 original articles that analyzed data from the Brazilian Study of Cardiovascular Risks in Adolescents (ERICA). Although Brazil has extensive experience in conducting national surveys, ERICA impresses not only by the size of the survey − approximately 75,000 adolescents were evaluated in 1,248 schools of 121 municipalities − but also by the coverage in all Brazilian territory and by the number of assessed risk factors. The sampling frame of the study allows national and regional estimates from capitals and surroundings of the five regions and also municipalities with more than 100 thousand inhabitants^5^.

Considering that more than 80.0% of Brazilian adolescents attend school, the school base was chosen to define ERICA’s population sample. Although searching schools is easier than searching households, to obtain students presentative sample is a complex task. The sampling plan of the study solved this complexity, because it guaranteed representation for small and big municipalities in the national and regional level, and for each capital. Detailed description of the sample can be found in Vasconcellos et al.^5^

The survey assessed important aspects of adolescent health, including mental health and behaviors usually initiated at that stage of life, such as smoking, alcohol consumption and sexual life.

The research updates adolescents’ food consumption data, which was performed for the first time in Brazil in 2008-2009, in a food consumption survey added to the Household Budget Survey (HBS). ERICA, used the same evaluation method from the national survey. The method was improved with the inclusion of new items and portion sizes allowing the estimation of the amount of calories, nutrients and micronutrients consumed individually using statistical techniques, based on replication applied to a subsample of the population.

ERICA was initiated in 2008, when the Ministry of health (SCTIE/DECIT) launched a public call to select scientific institutions for the development of a national epidemiological investigation on metabolic syndrome in adolescents. The project ERICA of the Instituto de Estudos em Saúde Coletiva of the Universidade Federal do Rio de Janeiro was selected. Then, a large national network of researchers from different areas of health involved with adolescent health, cardiovascular diseases, and obesity, among others, was formed. Initially, 21 institutions signed the covenant and, subsequently, other 16 joined them. A center of local and central coordinations was formed with researchers and consultants participating in different stages of the study, such as the planning and the implementation of data analysis.

The main goal of ERICA was to estimate the prevalence of cardiovascular risk factors in adolescents from 12 to 17 years who attended public and private schools in Brazilian cities with more than 100 thousands inhabitants. The study will also enable the investigation of several associations involving sociodemographic characteristics, cardiovascular risk factors and metabolic changes.

Besides the questionnaire filled out by the adolescents through electronic data collection, weight, height, waist circumference, and blood pressure were measured. Also, in subsample of approximately 42.000 adolescents studying in the morning term, blood was drawn for measuring lipids, glucose, insulin, and glycated hemoglobin^4^. Food consumption was assessed using 24-hour dietary recall questionnaire^1^, with replication in non-consecutive days in a subsample. Information about family morbidity, birth weight of the adolescents and breastfeeding were obtained from a questionnaire sent to the students’ guardians.

The papers in this supplement describe the prevalence and the main factors associated with frequent disorders among Brazilian adolescents, such as metabolic syndrome, hypertension, obesity, dyslipidemia, smoking, physical inactivity, alcohol consumption, common mental disorder, asthma, healthy and unhealthy habits, and sexual behavior, as well as food intake. The broad spectrum presented by the articles that comprise the Supplement has value for both understanding the health of adolescents and to evaluate the government health policies aiming at the protection of that life cycle. In addition, knowing adolescent health has immediate and long-term consequences on the health of the population as a whole, since many risk factors studied tend to persist into adulthood.

The collection methods used, as well as the protocols, represent important methodological advances.^2^ Thus, the assessment of food consumption was accomplished through 24-hour recall questionnaire, using the multiple-pass method in data collection, with data analysis for statistical modeling, allowing better estimation of prevalence and population distributions. The development of a computer program for data collection of food consumption is one of the important advancements of the research.

Blood pressure was measured with digital monitor validated for adolescents and its use is much simpler than the mercury sphygmomanometers, which allowed more accurate and standardized measurement. The heterogeneity in the techniques of blood pressure measurement has been an obstacle to the comparability of studies that evaluate the arterial pressure. In Brazil, the small number of studies on hypertension in adolescents in some regions represents a gap to be filled with the information obtained on ERICA^3^.

Blood samples, considered by many to be infeasible due to logistic complexity and fear of unacceptable number of refusals, was held in schools without major complications. It reached almost 60.0% of adolescents’ participation, allowing an unprecedented analysis of the lipid profile and glucose metabolism of Brazilian adolescents. A biorepository was created with a subsample of serum aliquots of adolescents from three states – Ceara, Rio de Janeiro and Rio Grande do Sul – and Federal District, for analysis of inflammatory markers and new markers of cardiovascular risk factors.

Other results of ERICA with impact on the development of future research arise from the important exchanges between teaching and research institutions across the country. Partnerships allow interaction between these institutions in the formulation of strategies to prevent obesity, diabetes and cardiovascular risk factors, as well as other chronic diseases that share risk profile with cardiovascular diseases (sedentarism, smoking, alcoholism, obesity), e.g., neoplasms.

Results of the study emphasize the need of short term changes regarding availability and stimulation of healthier foods consumption, as well as activities and behaviors that reduce the exposure of these adolescents to obesity, sedentarism and smoking, among other cardiovascular risk factors.

Aligned with the results of other several national surveys, ERICA also points out to significant regional contrasts, which need to be better explored for more effective public health interventions. Thus, North and Northeast regions present the lowest prevalence of obesity, approximately 5.0%, while in the South and Southeast regions the prevalences are close to 10.0%. Variations with the same tendency occur for hypertension. Also, important variations in the prevalence of obesity between capital cities of the same region, as in Florianopolis, SC, and Porto Alegre, RS – the former presenting almost the double of prevalence when compared to the latter –, showing that the data set of ERICA provide a more accurate understanding on the distribution of the diseases evaluated and their risk factors.

To avoid a worsening quality and lower life expectancy of adolescents in relation to their parents, which will interrupt the continuous increase in life expectancy over the last 100 years^4^, it is necessary to evaluate and invest in strategies that effectively prevent obesity and other risk factors as well as their consequences, in early stages of life.
