# Supplementary material for: Study of Cardiovascular Risk Factors in Adolescents (ERICA): results and potentiality
Source: Rev Saude Publica. 2016 Feb 2;50(Suppl 1):2s. doi: 10.1590/S01518-8787.201605000SUPL1AP (PMC4767038; doi:10.1590/S01518-8787.201605000SUPL1AP)
Supplement: Translation

# Translation: Estudo dos Riscos Cardiovasculares em Adolescentes (ERICA): resultados e potencialidade

**Authors:** Katia Vergetti Bloch, Marly Augusto Cardoso, Rosely Sichieri

**Affiliations:** I Instituto de Estudos em Saúde Coletiva. Universidade Federal do Rio de Janeiro. Rio de Janeiro, RJ, Brasil; II Departamento de Nutrição. Faculdade de Saúde Pública. Universidade de São Paulo. São Paulo, SP, Brasil; III Departamento de Epidemiologia. Instituto de Medicina Social. Universidade do Estado do Rio de Janeiro. Rio de Janeiro, RJ, Brasil

Este suplemento apresenta 13 artigos originais que analisaram dados do Estudo dos Riscos Cardiovasculares em Adolescentes (ERICA). Embora o Brasil tenha grande experiência na condução de inquéritos de base nacional, o ERICA impressiona não só pelo tamanho da pesquisa − foram avaliados cerca de 75.000 adolescentes em 1.248 escolas de 121 municípios − mas também pela abrangência em todo território brasileiro e pelo número de fatores de risco avaliados. O estudo tem representatividade nacional, regional, de capitais e de estratos do interior das cinco regiões para municípios com mais de 100 mil habitantes^5^.

Considerando que mais de 80,0% dos adolescentes brasileiros frequentem escola, a base escolar da população amostral do ERICA pode ser considerada muito expressiva para seus propósitos. Porém, apesar de buscar escolas seja mais fácil que buscar domicílios, obter amostra representativa de escolares é uma tarefa complexa. O plano amostral do estudo atesta essa complexidade, pois garantiu representatividade para o conjunto de municípios de médio e grande porte no nível nacional e regional e para cada capital. A descrição pormenorizada da amostra encontra-se em Vasconcellos et al.^5^

A pesquisa buscou avaliar importantes aspectos da saúde do adolescente, incluindo a saúde mental e comportamentos, geralmente iniciados nessa fase de vida, como tabagismo, consumo de álcool e vida sexual.

A pesquisa atualiza, ainda, dados de consumo alimentar de adolescentes pesquisados pela primeira vez no Brasil, em 2008-2009, em inquérito alimentar adicionado à Pesquisa de Orçamentos Familiares (POF). O ERICA, utilizando método de avaliação de consumo, aprimorado a partir do inquérito nacional, permite estimar a quantidade de calorias, nutrientes e micronutrientes, consumidos individualmente, empregando técnicas estatísticas, com base na replicação da medida do consumo em parcela da população.

O ERICA foi iniciado em 2008 quando o Ministério da Saúde (SCTIE/DECIT) lançou uma chamada pública a fim de selecionar instituições científicas para desenvolvimento de inquérito epidemiológico nacional sobre síndrome metabólica em adolescentes. O projeto ERICA, do Instituto de Estudos em Saúde Coletiva da Universidade Federal do Rio de Janeiro, foi selecionado. Iniciou-se, a partir de então, a formação de uma grande rede nacional de pesquisadores de diferentes áreas da saúde envolvidos com saúde do adolescente, doenças cardiovasculares e obesidade, entre outras. Inicialmente, 21 instituições assinaram o convênio e, posteriormente, outras 16 juntaram-se a essas. Foi formado um núcleo de coordenação central e coordenações locais, com pesquisadores colaboradores e consultores participando em diferentes etapas do estudo, do planejamento e execução à análise dos dados.

O objetivo principal do ERICA foi estimar a prevalência de fatores de risco cardiovascular em adolescentes de 12 a 17 anos que frequentavam escolas públicas e privadas em cidades brasileiras com mais de 100 mil habitantes. O estudo permitirá também a investigação de inúmeras associações envolvendo características sociodemográficas, fatores de risco cardiovascular e alterações metabólicas.

Além de um questionário autopreenchido pelos adolescentes, por meio eletrônico de coleta de dados, foram aferidos: peso, altura, circunferência de cintura, pressão arterial. Ainda, em subamostra de cerca de 42.000 adolescentes que estudavam no turno da manhã, foi coletado sangue para dosagem de lipídios, glicose, insulina e hemoglobina glicada.^4^ O consumo alimentar foi avaliado utilizando-se inquérito recordatório alimentar de 24 horas,^1^ com replicação em dias não consecutivos em parcela da população. Informações sobre morbidade familiar, peso ao nascer do adolescente e amamentação foram obtidas por meio de questionário enviado pelos alunos aos responsáveis.

Os artigos deste Suplemento descrevem as prevalências e os principais fatores associados a distúrbios frequentes entre adolescentes brasileiros, tais como síndrome metabólica, hipertensão arterial, obesidade, dislipidemia, tabagismo, inatividade física, consumo de álcool, transtorno mental comum, comportamento alimentar saudável e não saudável e asma, caracterizando também práticas alimentares e comportamento sexual. O amplo panorama apresentado pelos artigos que compõem este Suplemento tem valor tanto para a compreensão da saúde dos adolescentes brasileiros, como para as políticas de saúde do governo visando à proteção desse ciclo de vida. Além disso, conhecer a saúde do adolescente tem consequências imediatas e de longo prazo na saúde da população como um todo, uma vez que vários fatores de risco estudados tendem a se manter na vida adulta.

Os métodos de coleta utilizados, bem como os protocolos, representam avanços metodológicos importantes^2^. Assim, a avaliação do consumo alimentar foi realizada por meio de inquérito recordatório de 24 horas, utilizando-se o método de múltiplas passagens na coleta dos dados, com análise dos dados por modelagem estatística, permitindo melhor estimativa de prevalências e distribuições. O desenvolvimento de um programa de computador para coleta dos dados de consumo alimentar é um dos desdobramentos importantes da pesquisa.

A pressão arterial foi aferida com monitor digital validado para adolescentes e de uso muito mais simples que os esfigmomanômetros de mercúrio, permitindo aferição mais acurada e padronizada. A heterogeneidade nas técnicas de aferição de pressão arterial tem sido um obstáculo à comparabilidade de estudos que avaliam a pressão arterial. No Brasil, o pequeno número de estudos sobre hipertensão arterial em adolescentes em algumas regiões representa uma lacuna a ser preenchida com as informações obtidas no ERICA^3^.

A coleta de sangue, considerada por muitos inviável devido à complexidade logística a ao temor de número inaceitável de recusas, foi realizada nas escolas sem grandes intercorrências. Alcançou quase 60,0% de participação dos adolescentes, permitindo uma análise inédita do perfil lipídico e do metabolismo glicídico dos jovens brasileiros. Foi criado um biorrepositório com uma subamostra de alíquotas de soro dos adolescentes de três estados – Ceará, Rio de Janeiro e Rio Grande do Sul e Distrito Federal, para análise de marcadores inflamatórios e de novos marcadores de risco cardiovascular.

Outros resultados do ERICA com impacto no desenvolvimento de pesquisas futuras decorrem do importante intercâmbio entre instituições de ensino e pesquisa de todo o País. As parcerias permitem ainda interação entre essas instituições na formulação de estratégias de prevenção de obesidade, diabetes e fatores de risco cardiovascular, assim como de outras doenças crônicas que compartilham perfil de risco com doenças cardiovasculares (sedentarismo, tabagismo, alcoolismo, obesidade), por exemplo, as neoplasias.

Cabe salientar a necessidade de que os resultados produzam mudanças a curto prazo com relação à disponibilidade e estímulo ao consumo de alimentos mais saudáveis, bem como ao estímulo a atividades e comportamentos que reduzam a exposição desses adolescentes à obesidade, ao sedentarismo e ao tabagismo, entre outros fatores de risco cardiovascular.

Assim como várias pesquisas nacionais têm mostrado, os resultados do ERICA também apontam contrastes regionais importantes, que precisam ser melhor explorados para planejamento de intervenções em saúde pública mais efetivas. Assim, as regiões Norte e Nordeste apresentam as menores prevalências de obesidade, ao redor de 5,0%, enquanto nas regiões Sul e Sudeste as prevalências são próximas a 10,0%. Variações na mesma direção ocorrem para hipertensão arterial. Há, ainda, variações importantes da prevalência de obesidade entre capitais de uma mesma região, como em Florianópolis, SC, e Porto Alegre, RS – esta última apresentando quase o dobro da prevalência da primeira –, mostrando que o conjunto de dados do ERICA propiciará a compreensão mais acurada da distribuição das doenças avaliadas e de seus fatores de risco.

Para que os jovens de hoje não envelheçam com uma pior qualidade e menor expectativa de vida em relação aos seus pais, interrompendo o aumento contínuo da esperança de vida nos últimos 100 anos,^4^ é preciso avaliar e investir em estratégias que efetivamente previnam a obesidade e outros fatores de risco, e suas consequências, em estágios precoces da vida.
